# Case report: Tongdu Xingshen acupuncture for a patient with persistent vegetative state after herpes simplex virus encephalitis

**DOI:** 10.3389/fneur.2022.896721

**Published:** 2022-10-03

**Authors:** Bingxu Jin, Yuyuan Tang, Yunyun Wu, Zhenhuan Liu

**Affiliations:** ^1^Department of Rehabilitation, Panyu Hospital of Chinese Medicine, Guangzhou, China; ^2^Clinical Research and Big Data Laboratory, South China Research Center for Acupuncture and Moxibustion, Medical College of Acupuncture-Moxibustion and Rehabilitation, Guangzhou University of Chinese Medicine, Guangzhou, China; ^3^Medical College of Acupuncture-Moxibustion and Rehabilitation, Guangzhou University of Chinese Medicine, Guangzhou, China; ^4^Department of Children Rehabilitation, Nanhai Maternity and Children Hospital, Guangzhou University of Chinese Medicine, Foshan, China

**Keywords:** persistent vegetative state (PVS), herpes simplex virus encephalitis (HSVE), acupuncture, traditional Chinese medicine, case report

## Abstract

**Introduction:**

A persistent vegetative state (PVS) can be caused by traumatic or non-traumatic brain injury. PVS is a complex clinical condition with numerous complications. Nursing care, medical treatment, and comprehensive rehabilitation are necessary to improve the outcomes of PVS. However, the prognosis remains unsatisfactory. Acupuncture therapy has been used as a rehabilitation strategy to treat patients with PVS in China, showing better results in the recovery of consciousness, intellectual capability, and motor function.

**Case description:**

We present the case of a 4-month-long PVS after herpes simplex virus encephalitis (HSVE) in a 3.5-year-old boy who underwent Tongdu Xingshen acupuncture integrated with Western medicine and rehabilitation. The patient regained consciousness post-treatment. His intelligence and motor function gradually recovered after seven treatment sessions.

**Conclusion:**

Tongdu Xingshen acupuncture is a potential complementary therapy to optimize clinical outcomes in PVS.

## Introduction

Prolonged disorders of consciousness (PDoC) are defined as any disorder of consciousness that has continued for at least 4 weeks following sudden-onset brain injury. PDoC includes vegetative state/unresponsive wakefulness syndrome (VS/UWS) and minimally conscious state (MCS) (1). VS/UWS is defined as a state of unaware wakefulness in which there is a preserved capacity for spontaneous or stimulus-induced arousal—as evidenced by sleep–wake cycles and a range of reflexive and spontaneous behaviors.

Few studies have reported the epidemiology of VS. A systematic review reported that the prevalence of VS ranged from 0.2 to 6.1 patients with VS/UWS per 100,000 people (2). Studies on children found a prevalence rate of 6–80/million children (3).

The major causes of VS are trauma, vascular events, hypoxia or hypoperfusion, infection or inflammation, and toxic or metabolic disorders (1). Central nervous system infections account for 5–10% of pediatric persistent VS (PVS) (3). Viruses are responsible for 20–50% of all cases of encephalitis. Herpes simplex virus is the most common sporadic encephalitis worldwide (4). Herpes simplex virus encephalitis (HSVE) is fatal in more than 70% of patients if untreated. Antiviral treatment has decreased mortality to 20–30% (5), whereas most surviving patients continue to suffer from moderate-to-severe neurological sequelae including PVS. A previous study (6) reported that HSVE survivors experience amnestic difficulties (75%), global cognitive decline (25%), and personality and behavioral abnormalities (40–60%). It has also been reported that nearly 1% of pediatric patients are found to be in a VS at long-term follow-up evaluation (7, 8).

There are no established therapies for children with PDoC (9). Currently, the clinical evidence for therapies for children with PDoC is inadequate. Drugs such as amantadine, pramipexole, donepezil, and zolpidem are sometimes used in clinical practice (9, 10). Specialized neurological rehabilitation is recommended, and traditional Chinese medicine is used as a rehabilitation method (1). The prognosis for regaining consciousness and subsequent survival is poor, and long-term survival from PVS among the pediatric population is poor (11). Thus, the treatment of PDoC in the pediatric population deserves further research.

Here, we report the case of PVS after contracting HSVE in a young child. A combination of Tongdu Xingshen acupuncture therapy and Western medicine was adopted. The patient progressed favorably with respect to the level of consciousness and intelligent and motor function.

## Case presentation

A 3.5-year-old boy showed perturbed consciousness with movement and intellectual dysfunction after suffering from HSVE and secondary epilepsy (Supplementary Video 1). He was administered antiviral and antiepileptic therapy, as well as immunomodulatory and neurotrophic agents. On admission, he was unsteady with his head upright and was able to roll over but unable to sit up independently. He was unable to actively or passively grasp objects. Although he was able to open his eyes, no visual tracking was observed. He was unable to follow any instructions. His left limb had involuntary activity sometimes accompanied by altered sleep–awake cycles. He was able to cry and make a “hum” sound through his nose. He required bolus nasogastric tube feedings due to dysphagia. His growth and development had proceeded normally until the onset of HSVE. Seizures were under control after taking levetiracetam tablets (Supplementary Video 1).

On initial physical examination, his vital signs were normal. He presented deficits in upright head/neck control and inability to support himself with his elbows and hands. Although he could roll over, he had problems controlling the movement of all four limbs, making it difficult for him to crawl, stand, and walk independently. He could not easily control shifts in position, e.g., from lying down to sitting up. His muscle tone was generally normal, but limb weakness grade was 3/5 (MRC strength scale). Adductor angle, popliteal angle, and dorsiflexion angle of his foot was 150°, 150°, and 70°, respectively. Knee/Achilles jerk reflexes were normal. Ankle clonus was positive. Neither the Babinski sign nor the meningeal irritation sign was positive.

His PVS score (12) was 5 (command execution = 0, body movement = 2, eye movement = 1, emotional reactions = 1, swallow = 0, and speech = 1). Gesell Developmental Schedules (GDS) scores (13) indicated a severe defect [gross motor (developmental quotient (DQ) = 4.4, fine motor (DQ = 0), adaptive behavior (DQ = 0), language (DQ = 0), and personal–social behavior (DQ = 0)]. Infants-Junior High School Students' Social Development Screening Test (14) (coarse score = 1 and standardized score = 6) indicated a severe abnormality, suggestive of movement and intellectual disorder. Gross Motor Function Measure-88 (GMFM-88) (15) was as follows: A = 82.4, B = 0, C = 0, D = 0, and total score = 16.5, indicating significant retardation in GMF.

**Figure 1:**
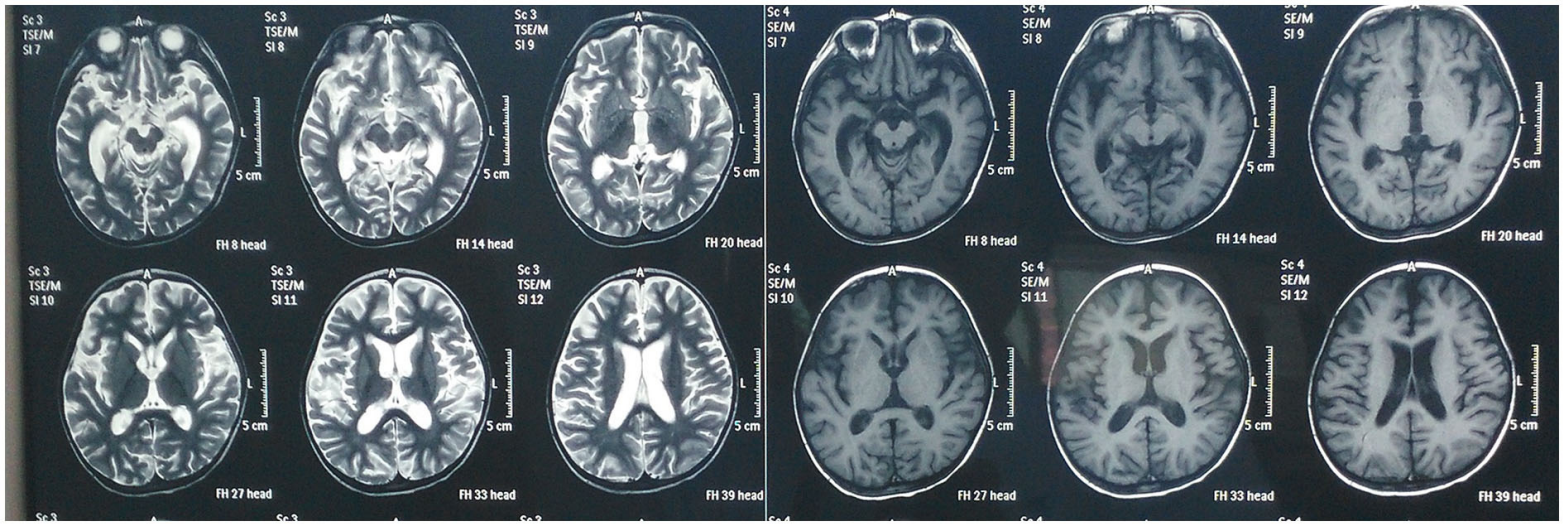
Magnetic resonance imaging of the brain before receiving Tongdu Xingshen acupuncture showed mild brain atrophy and pachymeningeal enhancement associated to meningitis.

Brain magnetic resonance imaging (MRI) showed that the sulcus in the bilateral cerebral hemispheres was slightly deeper than before, and the lateral ventricles were slightly enlarged. Some abnormal signals were observed around the bilateral ventricles. There were perivascular lesions in the bilateral frontal lobe and pachymeningeal enhancement (Figure 1). Video electroencephalogram (VEEG) demonstrated slow background activity. Numerous bilateral sharp waves and sharp wave complexes were observed in the posterior head and right rolandic area. Transcranial Doppler (TCD) indicated that the blood flow velocity of the bilateral middle cerebral artery was asymmetric, with its right side slightly slower. The anterior and posterior communicating arteries were unobstructed with compensatory capability. Visual evoked potential (VEP) and brainstem auditory evoked potential (BAEP) were normal.

The results of routine biochemical tests were normal. Immunologic tests found a high concentration of immunoglobulin G (IgG), while the concentration of IgA was decreased.

## Diagnosis

Because the age of onset of PVS was >1 year after birth and the patient showed a normal development before HSVE, cerebral palsy and inherited metabolic disease were ruled out. According to the nervous system, physical examination, and the disease being unprogressive, progressive muscular dystrophy was also ruled out.

The following diagnostic criteria for PVS (12), proposed at a meeting in Nanjing in April 1996, were applied: (1) no evidence of awareness of self or environment and inability to execute commands; (2) sufficiently preserved respiratory function and blood pressure; (3) intermittent wakefulness manifested by the presence of sleep–wake cycles; (4) no evidence of language comprehension or expression; (5)unconsciousness with eyes open; (6) no visual tracking; and (7) hypothalamic and brainstem autonomic functions sufficiently preserved to permit survival with medical and nursing care.

The patient's clinical presentations conformed to the abovementioned diagnostic criteria, and his PVS score of 5 indicated incomplete vegetative syndrome. VEEG activity demonstrated typically slow wave activity, and the patient had a history of HSVE. Therefore, he was diagnosed to be on PSV during convalescence from HSVE. Based on the case history, VEEG and MRI results, and medication history, a diagnosis of secondary epilepsy and brain atrophy was considered.

## Treatment and outcomes

### Routine treatment

The patient was given intravenous (IV) scopolamine (one time a day, 0.03–0.06 mg/kg; the IV was adjusted according to the patient's conditions in the first three treatment courses) compound Danshen tablets (two times a day with one tablet each time in the remaining four treatment courses) to improve brain microcirculation, and cattle encephalon glycoside and ignotin (CEGI) injection (one time a day, 2 ml, IV throughout the treatment period) to alleviate the nerve function injury. Regular rehabilitation therapy included exercise therapy, massage, and speech and cognitive training.

### Acupuncture therapy

The acupuncture method used was Tongdu Xingshen acupuncture comprising scalp acupuncture and body acupuncture (Supplementary Table S1).

#### Treatment course

In the first four treatment courses, the selected acupoints were nine intelligent needles [Sishencong (EX-HN1) plus forehead five needles], temporal three needles, BaiHui (GV20), foot motor sensory area, motor area, balance area, second speech area, spirit-emotion area, YinTang (EX-HN3), Neiguan (PC6), Sanyinjiao (SP6), and Shenmen (HT7).

In the remaining three courses, acupoints were adjusted based on the previous four courses. The foot motor sensory area, motor area, and spirit-emotion area were removed. Areas of the heart and liver were added during these treatment courses.

#### Acupuncture manipulations

Acupuncture treatment was performed by an independent certified practitioner (acupuncturist) with 5 years of clinical experience.

##### Scalp acupuncture and Bai Hui (GV 20)

Disposable stainless steel needles (size 0.30 mm × 40 mm; Huatuo, Suzhou Medical Appliance, Suzhou, Jiangsu Province, China) were manually inserted at an angle of ~15° to a depth of 20–35 mm. For a total of 120 min, the needles were twirled and rotated at 180–200 revolutions/min for 3 min every 30 min.

##### Body acupuncture

a. *Yin Tang*: The 0.30 mm × 25 mm acupuncture needles were inserted obliquely in the direction of the nasal root at an angle of ~10–20° and an insertion depth of 10–15-mm.

b. *Bilateral Neiguan and Shenmen*: The needles were vertically thrust at depths of 10–15 (Neiguan) and 8–10 mm (Shenmen).

c. *Bilateral Sanyinjiao*: The 0.3 mm × 40 mm needles were vertically inserted at a depth of 15–20 mm.

d. *Manipulation*: The even reinforcing-reducing method was adopted by twirling the needles for at least 180 revolutions/min for 3 min every 10 min. The needle retention time was 30 min. “De qi” is an indication of effective needling. Acupuncturists will feel tightness around the needle when the qi arrives.

e. *Treatment course*: The patient received seven courses of acupuncture with an average of 19 days each course. Acupuncture was implemented every other day for 10 times per treatment course. If the patient caught a cold or other conditions that influenced acupuncture treatment, the course was prolonged.

**Figure 2:**
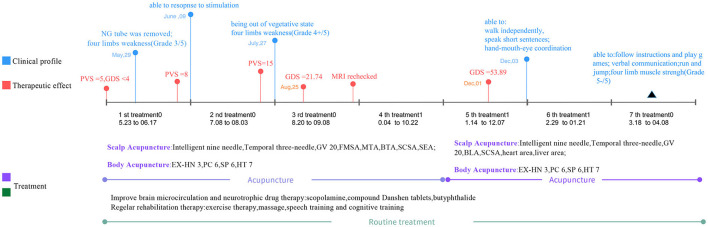
The timeline of diagnosis and treatment for this patient. NG tube, nasogastric tube; PVS, persistent vegetative state; GDS, Gesell Developmental Schedules; MRI, magnetic resonance imaging; FMSA, Foot motor sensory area; MTA, Motor area; BLA, Balance area; SCSA, Second speech area; SEA, Spirit-emotion area; GV 20, Baihui acupoint; EX-HN 3, Yintang acupoint; PC 6, Neiguan acupoint; HT 7, Shenmen acupoint; SP 6, Sanyinjiao acupoint.

The diagnosis and treatment process is illustrated in Figure 2.

### Evaluation of therapeutic effect

#### PVS score scale

The curative effect was evaluated using a PVS score scale proposed at the meeting in Nanjing in April 1996 (12). The total score was calculated according to the sum of scores for six clinical features. The standard for the total score is as follows: complete vegetative state (PVS ≤ 3); incomplete vegetative syndromes (4 ≤ PVS ≤ 7); transitional vegetative syndromes (8 ≤ PVS ≤ 9); out-of-vegetative-state (10 ≤ PVS ≤ 11); and recovery of consciousness (PVS ≥ 12). A patient is in the out-of-vegetative-state if he can execute instructions.

#### Gesell developmental schedules

The Chinese version of the GDS (13) is used to evaluate neurodevelopmental symptoms in children, such as gross motor skills, fine motor skills, adaptability, language, and personal–social activity. The degree of mental development is classified according to the average DQ score: normal (DQ ≥6), borderline (DQ: 76≤ –≤85), mild defect (DQ: 55 ≤-≤ 75), moderate defect (DQ: 40≤-≤54), and severe and extremely severe defect (DQ ≤ 39).

### Therapeutic effect

**Figure 3:**
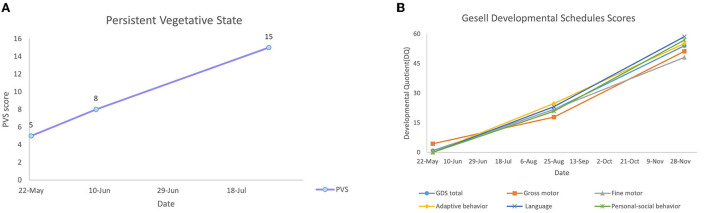
The therapeutic effect on PVS **(A)** and GDS score **(B)** of patient.

Approximately 1 week after the abovementioned treatment, the nasogastric tube was removed, and the patient was fed thick porridge and rice cereal. After 12 days of treatment, the patient smiled on his own. After completion of the first treatment course, the PVS score on the PVS rating scale increased to 8 (command execution = 0, body movement = 2, eye movement = 1, emotional reactions = 2, swallow = 2, and speech = 1) (Figure 3), indicating that the patient evolved favorably. The patient showed the ability to control his head and neck when he was upright and could sit independently. Muscle strength in his upper limbs gradually recovered, and he demonstrated his ability to support the upper body with his hands and elbows. He had a normal crying and laughing reaction to stimulation (Supplementary Video 2).

At the end of the second treatment course, the patient was out of VS (the PVS rating scale was 15) (command execution = 2, body movement = 3, eye movement = 3, emotional reactions = 3, swallow = 2, and speech = 2) (Figure 3). He was able to perform simple tasks with continuous eye tracking and eye contact. He could speak simple words like “mum” and “dad,” sit up straight, stand up, and walk slowly by holding or touching a support surface. The grip strength of his hand increased. The urge to defecate gradually recovered. Four limbs muscle strength was gradually recovered (grade 4+/5) (Supplementary Video 3).

**Figure 4:**
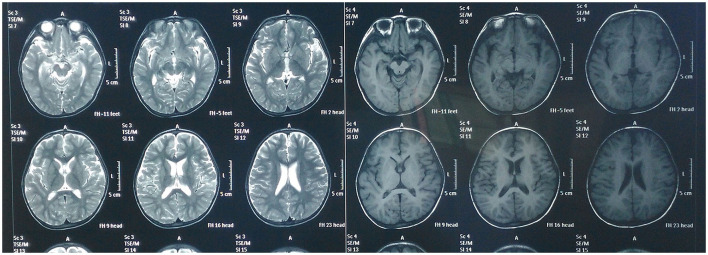
Magnetic resonance imaging of the brain after receiving Tongdu Xingshen acupuncture suggested mild brain atrophy and pachymeningeal enhancement associated to meningitis improved.

In the third treatment course, GDS was assessed (Figure 3). The score was 21.74, indicating a severe and extremely severe defect [gross motor (DQ = 17.9), fine motor (DQ = 21.9), adaptive behavior (DQ = 24.8), language (DQ = 23.2), and personal–social behavior (DQ = 20.9)]. MRI was rechecked after this course, demonstrating that mild atrophy-like changes in the bilateral cerebral hemispheres improved and the abnormal signal near the bilateral ventricles was basically absorbed. The abnormal signal in the white matter of bilateral frontal lobes indicated the possibility of poor myelination, and a follow-up review is advised to rule out focal demyelination. Bilateral temporal dura enhanced, indicating intracranial infection sequelae, for which short-term review is recommended (Figure 4).

After five treatment courses, the patient was able to resume a full diet. He could sit and walk independently and grasp objects flexibly. He demonstrated hand–mouth–eye coordination. He was able to communicate with others by talking short sentences of 5–6 words and could play a simple imitation game. GDS scores increased to 53.98, suggesting a moderate defect [gross motor (DQ = 51.2), fine motor (DQ = 48.1), adaptive behavior (DQ = 55.1), language (DQ = 58.6), and personal–social behavior (DQ = 56.9)] (Figure 3) (Supplementary Video 4).

In the last treatment course, the patient was able to follow instructions and play games. He exhibited fluency in routine verbal communication. He could not only sit, stand, and walk independently, but also run and jump. He exhibited hand–mouth–eye coordination and was able to stand upright independently. Muscle strength in all four limbs was generally normal. Ankle clonus was positive (Supplementary Video 5). No adverse or unanticipated events were reported throughout the treatment period. At the 1-year follow-up examination, ambulatory electroencephalography (AEEG) showed a paroxysmal complex of sharp–slow waves in bilateral brain areas. MRI demonstrated few bilateral lacunar ischemic foci in the subfrontal cortical white matter. The patient was studying in kindergarten and was able to actively communicate with other children and teachers.

### Preventive measures during acupuncture

To avoid unexpected situations, the acupuncturists selected a comfortable posture for the needling and paid due attention to the manipulation. Parents were asked to carefully observe the patient during acupuncture to monitor for emergency conditions. If any adverse event occurred, appropriate measures were taken immediately.

## Discussion

Evidence has shown that some of the factors involved in the prognosis of VS/UWS are etiology, age at the time of acute injury, and time spent in the same state. As for age, the recovery of consciousness and survival rates are higher in younger patients than in older ones; however, pediatric patients <1 year of age have been reported to show higher mortality (16). Better consciousness and independence outcomes are observed in traumatic causes than in non-traumatic ones (17). The correlation between time spent in VS/UWS and a better outcome is negative. Given that VS/UWS impairs neurological development among pediatric patients, many patients require long-term care. Therefore, effective and early intervention is indispensable for the long-term prognosis of VS/VWS.

Acupuncture is commonly used in various neurological conditions. The addition of acupuncture can alleviate consciousness disorders. It is reported that the addition of acupuncture can remarkably promote the recovery of the consciousness level (18) and improve motor function of the limbs. Further, computed tomography (CT) demonstrated a reduction in the width of the third ventricle (19) and apparently reduced the mean curing time (20). Only a few adverse events were reported. The abovementioned studies suggested that the addition of acupuncture is safe and effective.

Scalp acupuncture is a modern acupuncture technique that combines the traditional needling method with modern medical knowledge (21). It has been widely used to treat cerebral diseases in traditional Chinese medicine.

The mechanism underlying scalp acupuncture therapy in cerebral diseases remains elusive. However, the following are considered potential mechanisms: (1) increases cerebral blood flow; (2) improves cerebral oxygen metabolism; (3) reduces the deterioration of brain tissues as a result of free radicals and inflammatory factors (22); (4) enhances synaptic plasticity *via* the regulation of neurotrophic factors (23); and (5) regulates the brain microenvironment *via* nerve growth-related proteins (24).

Tongdu Xingshen acupuncture is developed based on Lin's scalp acupuncture, Jiao's scalp acupuncture, and our clinical practice. Our previous studies reported that Tongdu Xingshen acupuncture improved intelligence, language ability, social adaptive ability, and motor function (25–29). It exerts stimulation on the corresponding scalp projection area of the cerebral cortex including the frontal lobe (precentral gyrus), parietal lobule (postcentral gyrus), paracentral lobule, temporal lobe (posterior superior temporal gyrus), and acerebellar hemisphere. The effect of acupuncture transmitted through the cortical–thalamic–cortical pathway thereby positively modulates the corresponding areas of memory, the language center, the motor center, or balance. As for the retention time of the needle, a curative effect is positively associated with the length of the retention time. Retaining needles for 2 h is optimal for intellectual and gross motor development in children with cerebral palsy based on our previous research (30).

According to the basic theory of traditional Chinese medicine, the location of the disease in VS is the brain. The brain is made up of “marrow.” It has been recognized that the functions of the sense organs and body motion are linked with the brain in *Errors on Medicine Corrected* (*Yilin Gai Cuo*). The governing vessel has a close relationship with the brain, spinal marrow, and kidneys. Tongdu Xingshen acupuncture mainly stimulates the governing vessel to nourish the brain *via* ascending Yang Qi and enriching the marrow beneficial for the recovery of consciousness and nerve repair.

This case report has some limitations. First, the latest version of the diagnostic criteria and clinical efficacy scales for PVS were not adopted, but we propose that this did not influence diagnostic accuracy. Furthermore, intervention adherence and tolerability were not accessed. Nevertheless, the patient and his parents strictly followed the treatment routine and were hospitalized on time, suggesting good intervention adherence and tolerability.

In this case presentation, a 3.5-year-old boy with PVS after HSVE recovered from VS, and his cognitive and motor function generally improved after receiving a combination of Tongdu Xingshen acupuncture and modern medicine. This showed that acupuncture as an adjunctive therapy is effective in the recovery stage of PVS. It is recommended that patients with PVS receive acupuncture therapy as soon as possible when vital signs become stable. Acupoints located on the head are frequently used to stimulate the scalp projection area of each brain functional area. Earlier application of acupuncture is associated with a higher probability of awakening and fewer neurological sequelae.

### Patient's perspective

We sought medical help from Chinese medicine after receiving Western medicine therapy. Considering that the conditions were complex, we attempted to receive acupuncture in combination with routine treatment. After a week-long treatment, our son was able to swallow thick porridge or rice cereal. This favorable turnaround gave us confidence in the current treatment plan. The medical staff were patient and attentive. Throughout the therapy session, we were delighted to see his progress.

## Data availability statement

The original contributions presented in the study are included in the article/Supplementary material, further inquiries can be directed to the corresponding author/s.

## Ethics statement

Written informed consent was obtained from the minor(s)' legal guardian/next of kin for the publication of any potentially identifiable images or data included in this article.

## Author contributions

BJ contributed to data collection, the design of the case report design, data analysis, and interpretation. YT contributed to the design of the case report, writing the initial and subsequent drafts after they were revised by all involved authors, and submitting the final case report. YW contributed to data analysis and interpretation. ZL contributed to data analysis and interpretation, as well as critically revising this paper. All authors contributed to the article and approved the submitted version.

## Conflict of interest

The authors declare that the research was conducted in the absence of any commercial or financial relationships that could be construed as a potential conflict of interest.

## Publisher's note

All claims expressed in this article are solely those of the authors and do not necessarily represent those of their affiliated organizations, or those of the publisher, the editors and the reviewers. Any product that may be evaluated in this article, or claim that may be made by its manufacturer, is not guaranteed or endorsed by the publisher.

## References

[1] Turner-Stokes L Wade D Playford D. Prolonged Disorders of Consciousness Guidelines. London (2020) (accessed April 03, 2020).

[2] van Erp WS Lavrijsen JC van de Laar FA Vos PE Laureys S Koopmans RT. The vegetative state/unresponsive wakefulness syndrome: a systematic review of prevalence studies. Eur J Neurol. (2014) 21:1361–8. 10.1111/ene.12483 25039901

[3] Ashwal S. The persistent vegetative state in children. Adv Pediatr. (1994) 41:195–222. 7992684

[4] Jorgensen LK Dalgaard LS Ostergaard LJ Norgaard M Mogensen TH. Incidence and mortality of herpes simplex encephalitis in denmark: a nationwide registry-based cohort study. J Infect. (2017) 74:42–9. 10.1016/j.jinf.2016.09.004 27717782

[5] Saraya AW Wacharapluesadee S Petcharat S Sittidetboripat N Ghai S Wilde H. Normocellular Csf in herpes simplex encephalitis. BMC Res Notes. (2016) 9:95. 10.1186/s13104-016-1922-9 26879928 PMC4753680

[6] Hokkanen L Launes J. neuropsychological sequelae of acute-onset sporadic viral encephalitis. Neuropsychol Rehabil. (2007) 17:450–77. 10.1080/09602010601137039 17676530

[7] Chen T Liu G. Long-term outcome of acute central nervous system infection in children. Pediatr Investig. (2018) 2:155–63. 10.1002/ped4.12054 32851253 PMC7331314

[8] Fowler A Stodberg T Eriksson M Wickstrom R. Long-term outcomes of acute encephalitis in childhood. Pediatrics. (2010) 126:e828–35. 10.1542/peds.2009-3188 20876179

[9] Giacino JT Katz DI Schiff ND Whyte J Ashman EJ Ashwal S. Practice guideline update recommendations summary: disorders of consciousness: report of the guideline development, dissemination, and implementation subcommittee of the American Academy of Neurology; the American Congress of Rehabilitation Medicine; and the National Institute on Disability, Independent Living, and Rehabilitation Research. Neurology. (2018) 91:450–60. 10.1212/WNL.0000000000005926 30089618 PMC6139814

[10] Group Group of Disorders of Consciousness and Conscious-promotion PCo Association NoCMD. Neurorepair of Chinese medical doctor association, diagnoses and treatments of prolonged disorders of consciousness: an experts consensus. Chin J Neuromed. (2020) 19:977–82. 10.3760/cma.j.cn115354-20200701-00525

[11] Ashwal S Eyman RK Call TL. Life expectancy of children in a persistent vegetative state. Pediatr Neurol. (1994) 10:27–33. 10.1016/0887-8994(94)90063-9 8198669

[12] Zhang G Wang Ch Xinsheng D. Diagnostic criteria and scoring scale for persistent vegetative state in China. Chin J Crit Care Med. (1999) 19:59–60.

[13] Song J Zhu Y. Children's Neuropsychological Tests. Shanghai: Shanghai Scientific and Technological Publishing Company (1987).

[14] Shufen L Bilu H Zhenwu L Guangying C Kai C Zhen L. Evaluation of the effect of intelligence screening on infant-junior high school students' social life ability scale. West China Med. J. (1993):15–7.

[15] Tao W Lu Z Wen F. The Influence of neurodevelopmental treatment on transforming growth factor-beta1 levels and neurological remodeling in children with cerebral palsy. J Child Neurol. (2016) 31:1464–7. 10.1177/0883073816656402 27364738

[16] Ashwal S. Recovery of consciousness and life expectancy of children in a vegetative state. Neuropsychol Rehabil. (2005) 15:190–7. 10.1080/09602010443000281 16350962

[17] Monti MM Laureys S Owen AM. The vegetative state. BMJ. (2010) 341:c3765. 10.1136/bmj.c3765 20679291

[18] Bao YC Zhang F Li Q Liu M Cheng XR Zhang YB. [Xingnao Kaiqiao acupuncture on promoting wake-up of vegetative state after brain injury]. Zhongguo Zhen Jiu. (2021) 41:1225–8. 10.13703/j.0255-2930.20201101-k0002 34762375

[19] Gao YL Xu GJ Zhu JG Peng G Xi J Xue MX. [Xiao's “Xingnaofusu” needling for regaining consciousness and the influence on width of the third ventricle in patients with persistent vegetative state]. Zhen Ci Yan Jiu. (2020) 45:233–6. 10.13702/j.1000-0607.190543 32202716

[20] Tang Y Shang Q Zhou LH. [Vegetative state treated with acupoint injection combined with plum-blossom needle in children: a randomized controlled trial]. Zhongguo Zhen Jiu. (2014) 34:421–5. 10.13703/j.0255-2930.2014.05.013 25022107

[21] Lee GE Lee PT Ran N Zhou J. Scalp acupuncture for children with cerebral palsy: a protocol for a systematic review. Medicine. (2019) 98:e18062. 10.1097/MD.0000000000018062 31770222 PMC6890304

[22] Wang S Liu K Wang Y Wang S He X Cui X. A proposed neurologic pathway for scalp acupuncture: trigeminal nerve-meninges-cerebrospinal fluid-contacting neurons-brain. Med Acupunct. (2017) 29:322–6. 10.1089/acu.2017.1231 29067143 PMC5653342

[23] Lin R Li X Liu W Chen W Yu K Zhao C. Electro-acupuncture ameliorates cognitive impairment via improvement of brain-derived neurotropic factor-mediated hippocampal synaptic plasticity in cerebral ischemia-reperfusion injured rats. Exp Ther Med. (2017) 14:2373–9. 10.3892/etm.2017.4750 28962170 PMC5609168

[24] Wang Z Fan X Chen K Yu X Gao J. Effects of three kinds of head Acupuncture therapies on regulation of brain microenvironment and rehabilitation of nerve function in rats with cerebral palsy. J Tradit Chin Med. (2021) 41:276–83. 10.19852/j.cnki.jtcm.2021.02.007 33825408

[25] Yili Z Bingxu J Wenjian Z Xuguang Q Suping L Zhenhuan L. intelligence nine needling therapy for children with cerebral palsy accompanied developmental delay. China Health Care Nutr. (2012) 22:2465–6. 10.3969/J.ISSN.1004-7484(X).2012.08.066

[26] Jin B Fu W Li N Xin Z Liu C. [Study of optimal parameters of scalp electroacupuncture for rehabilitation effect on children of cerebral palsy]. Zhongguo Zhen Jiu. (2018) 38:143–7. 10.13703/j.0255-2930.2018.02.009 29473356

[27] Qian X Jin B Zhang Y Zhao W Zhao Y Fu W. [Effects of scalp acupuncture on brain injury in premature infants with different months of age]. Zhongguo Zhen Jiu. (2018) 38:723–6. 10.13703/j.0255-2930.2018.07.011 30014666

[28] Zhenhuan L editor. Clinical study on the treatment of developmental delay in children with intelligence nine needles. In: The Second World Congress of Integrative Medicine Beijing: 2nd World Integrative Medicine Congress (2002).

[29] Li N Jin BX Li JL Liu ZH. [Treatment of autism with scalp acupunctur]. Zhongguo Zhen Jiu. (2011) 31:692–6. 10.13703/J.0255-2930.2011.08.002 21894689

[30] Jin B Fu W Qian X Liu Z Zhang Y. Clinical efficacy study of different scalp acupuncture schemes based on orthogonal design for cerebral palsy. Chin J Rehabil Med. (2015) 30:591–3. 10.3969/j.issn.1001-1242.2015.06.016

