# Supplementary material for: Case report: Tongdu Xingshen acupuncture for a patient with persistent vegetative state after herpes simplex virus encephalitis
Source: Front Neurol. 2022 Oct 3;13:896721. doi: 10.3389/fneur.2022.896721 (PMC9576148; doi:10.3389/fneur.2022.896721)
Supplement: Supplementary file 1 [file Table_1.DOCX]

Supplementary Table 1 Tongdu Xingshen acupuncture acupoints

| Acupoint | Location | Function | Needle |
| --- | --- | --- | --- |
| Intelligent nine needles(1) | Sishencong(EX-HN 1):A group of four points on vertex of head which are located 1 cun anterior, posterial and bilateral to Baihui (GV 20) | Resuscitation ,improving intelligence, refreshing the mind and strengthening  memory | 0.30 mm× 40 mm |
|  | Forehead five-needle :A total of 5 needles were punctured from front to back at a distance of 2cm above the forehead hairline, between the surface marks of the left and right sylvian fissures, and the 5 needles were equally spaced in a fan-shaped arrangement. |  |  |
| Motor area(MA)(2) | A line connecting 2 points called the upper and lower points of the Motor Area. The upper point is situated on the antero-posterior midline, 0.5 cm behind its midpoint. The lower point is the point in the temporal region where the supercilio-occipital line intersects the anterior hairline. The whole area line is divided into five  equal parts, and grouped with three sections, upper one-fifth, middle two-fifths and lower two-fifths. | Motor dysfunction |  |
| Foot motor sensory area(FMSA)(2) | Parallel to and 1 cm lateral to the anterior-posterior line. The line is 3 cm long and starts 1 cm posterior to the line representing the sensory area. | Paralysis, poor memory |  |
| Balance area(BLA)(2) | A line 4 cm long drawn downwards and parallel to the anterior-posterior midline from a point at the level of the external occipital protuberance, 3.5 cm lateral to the midline | Loss of balance due to cerebellar disorders |  |
| Second speech area(SCSA)(2) | A vertical line 3 cm long, parallel to the anterior-posterior midline, its  upper end 2 cm posterior-inferior to the parietal tubercle | Nominal aphasia |  |
| Spirit-emotion area(SEA)(2) | 2 cm side of the antero-posterior midline, a 4 cm line from Vasomotor Area to front | Emotion disorders |  |
| Heart area(2) | A line 2 cm long drawn directly backwards and parallel to the anterior-posterior midline from a point on the anterior hairline vertically above the right pupil of the eye | Emotion disorders |  |
| Liver area(2) | A line 2 cm long drawn directly backwards and parallel to the  anterior-posterior midline from a point on the anterior hairline vertically above the left pupil of the eye | Emotion disorders |  |
| Temporal three needle(3) | The temporal I needle is located on the about 1cm anterior to the inferior border of the parietal tubercle; the temporal II needle is located on 1.5cm above the tip of the ear; temporal Ⅲ needle is located on 2cm behind the point located on 2cm below the tip of the ear. | Intellectual disorders, memory impairment and aphasia |  |
| Bai hui (GV 20)(4) | at the midpoint of the line connecting the apexes of the two auricles | Coma, apoplexy, insomnia, hemiplegia, aphasia, manic psychosis | 0.30 mm × 25 mm |
| Yintang(EX-HN 3)(4) | At the forehead, at the midpoint between the two medial ends of the eyebrow | Coma, insomnia |  |
| Neiguan(PC 6)(4) | On the palmar aspect of the forearm, 2 cun above the transverse crease of the wrist, on the line connecting PC 3 and PC 7, between the tendons of m. palmaris longus and m. flexor carpi radialis | Paralysis, epilepsy, mental disorders |  |
| Shenmen(HT 7)(4) | On the wrist, at the ulnar end of the transverse crease of the writs, in the depression on the radial side of the tendon m. flexor carpi ulnaris | Insomnia, amnesia, Mania, epilepsy |  |
| Sanyinjiao(SP 6)(4) | On the medial aspect of the lower leg, 3 cun above the medial malleolus, on the posterior border of the medial aspect of the tibia | Improving motor function of lower limbs | 0.3 mm × 40 mm |

Reference

1. Yili Z, Bingxu J, Wenjian Z, Xuguang Q, Suping L, Zhenhuan L. Intelligence Nine Needling Therapy for Children with Cerebral Palsy Accompanied Mental Retardation *China Health Care & Nutrition* (2012) 22(14):2465-6.

2. Wang T. Acupuncture for Brain--Treatment for Neurological and Psychological Disorders. *The Journal of Chinese Medicine* (2021).

3. Jiuwei W, editor. Introduction to Lin's Scalp Acupuncture. *Sleep Disease Clinical and Related Basic Research Academic Exchange Conference and Continuing Education Training Course*; 2014; Shanghai,China.

4. Pacific WHOROftW. *Who Standard Acupuncture Point Locations in the Western Pacific Region*: World Health Organization (2008).
